# Genetic Association Studies of *MICB* and *PLCE1* with Severity of Dengue in Indonesian and Taiwanese Populations

**DOI:** 10.3390/diagnostics13213365

**Published:** 2023-11-01

**Authors:** Imaniar Noor Faridah, Haafizah Dania, Rita Maliza, Wan-Hsuan Chou, Wen-Hung Wang, Yen-Hsu Chen, Dyah Aryani Perwitasari, Wei-Chiao Chang

**Affiliations:** 1 Department of Clinical Pharmacy, School of Pharmacy, College of Pharmacy, Taipei Medical University, Taipei 11031, Taiwan; imaniar.faridah@pharm.uad.ac.id (I.N.F.); ocean.chou@tmu.edu.tw (W.-H.C.); 2 Department of Pharmacology and Clinical Pharmacy, Faculty of Pharmacy, University of Ahmad Dahlan, Yogyakarta 55164, Indonesia; haafizah@pharm.uad.ac.id; 3 Biology Department, Faculty of Mathematics and Natural Sciences, Andalas University, Padang 25175, Indonesia; ritamaliza@sci.unand.ac.id; 4 School of Medicine, College of Medicine, National Sun Yat-sen University, Kaohsiung 80424, Taiwan; bole0918@gmail.com; 5 Division of Infectious Disease, Department of Internal Medicine, Kaohsiung Medical University Hospital, Kaohsiung Medical University, Kaohsiung 80708, Taiwan; 6 Master Program for Clinical Pharmacogenomics and Pharmacoproteomics, School of Pharmacy, Taipei Medical University, Taipei 11031, Taiwan; 7 Integrative Research Center for Critical Care, Wan Fang Hospital, Taipei Medical University, Taipei 11696, Taiwan; 8 Department of Pharmacy, Wan Fang Hospital, Taipei Medical University, Taipei 11696, Taiwan

**Keywords:** severe dengue, *MICB*, *PLCE1*, single-nucleotide polymorphism (SNP)

## Abstract

Dengue is an arboviral disease that has spread globally and become a major public health concern. A small proportion of patients may progress from symptomatic dengue fever (DF) to dengue hemorrhagic fever (DHF) or dengue shock syndrome (DSS). Findings from a previous genome-wide association study (GWAS) demonstrated that variations in the major histocompatibility complex (MHC) class I chain-related B (*MICB*) and the phospholipase C epsilon 1 (*PLCE1*) genes were related to DSS in a Vietnamese population. This study investigated associations of variations in *MICB* (rs3132468) and *PLCE1* (rs3740360, rs3765524) with dengue severity and thrombocytopenia in both the Indonesian and Taiwanese populations. We sampled 160 patients from the Indonesian population and 273 patients from the Taiwanese population. None of the patients had DSS in the Taiwanese population. Based on age demographics, we found that dengue is more prevalent among younger individuals in the Indonesian population, whereas it has a greater impact on adults in the Taiwanese population. Our results showed the association between *MICB* rs3132468 and DSS. In addition, an association was identified between *PLCE1* rs3740360 and DHF in secondary dengue in Indonesian patients. However, there is no association of *MICB* or *PLCE1* variants with thrombocytopenia. This study highlights the value of genetic testing, which might be included in the clinical pathway for specific patients who can be protected from severe dengue.

## 1. Introduction

Dengue is an acute febrile illness caused by dengue virus (DENV) transmission. There are four serotypes of DENV, named DENV-1, DENV-2, DENV-3, and DENV-4. The incidence of dengue has dramatically risen in recent decades. Higher numbers of cases have been recorded in Brazil, Vietnam, the Philippines, Indonesia, India, Malaysia, and Pakistan [1,2,3]. Indonesia has a tropical climate that is favorable for DENV transmission. A previous study confirmed the correlation between the incidence rates of dengue and rainfall in Indonesia. Since rainfall occurs more frequently from January to April, the humidity is increased and there are higher chances of mosquitoes reproducing [4]. Four serotypes of DENV are known to be circulating in Indonesia simultaneously, although the predominance of the various DENV serotypes differs between cities [5]. The clinical manifestations range from asymptomatic dengue to dengue fever (DF) and dengue hemorrhagic fever (DHF), which includes dengue shock syndrome (DSS) [6]. The incidence rate of DHF in Indonesia is increasing significantly with a cyclic pattern and peaks every 6 to 8 years [7]. In Yogyakarta, the number of dengue cases in 2019 was 3399, with 7 reported deaths [8]. In 2015, Taiwan experienced a notable dengue fever outbreak impacting more than 40,000 individuals [9]. Subsequently, there was no outbreak in Taiwan until 2023, when a dengue fever epidemic emerged in the southern region, leading to several thousand confirmed cases.

Risk factors identified for severe types of dengue (DHF and DSS) include female gender, younger age in children, older age in adults, secondary dengue infection, and preexisting comorbidities such as diabetes mellitus and hypertension [10,11]. Several clinical signs and symptoms are also associated with progression to severe dengue, such as vomiting, abdominal pain, the presence of clinical fluid accumulation, a low platelet count, severe liver damage, intestinal hemorrhage, and a low level of serum albumin [10,11].

As dengue is a multifactorial disease, genetic polymorphisms may have significant correlations with severe dengue through relationships with the host immune system. Results from a previous genome-wide association study (GWAS) identified two genes that are significantly associated with DSS. These two DSS-susceptibility genes are the major histocompatibility complex (MHC) class I chain-related B (*MICB*) rs3132468 in the chromosome 6 region and the phospholipase C epsilon 1 (*PLCE1*) rs3765524 and rs3740360 on chromosome 10 [12]. A previous replication study confirmed the association of *MICB* rs3132468 and *PLCE1* rs3765524 with DSS in Thai patients [13]. Another study revealed that *MICB* rs3132468 and *PLCE1* rs3740360 are significantly associated with clinical phenotypes of dengue less severe than DSS [14]. The *MICB* gene encodes a ligand for the NKG2D type II receptor, to activate NK cells or innate lymphocytes as an early immune response during viral infection [15,16]. Meanwhile, PLCE1 might contribute to vascular leakage by regulating the Rho family of GTPase [17,18]. Apart from these diseases, associations of the *MICB* and *PLCE1* genes with the clinical outcomes of infectious diseases remain unclear. One study showed no association of *MICB* and *PLCE1* genotypes with the level of viremia or clinical features such as platelet level, minimum white cell count, and maximum hematocrit [19].

Considering the potential roles of the *MICB* and *PLCE1* genes in DENV infections, this study aimed to determine the effects of genetic polymorphisms of *MICB* rs3132468 and *PLCE1* rs3740360 and rs3765524 on disease severity and thrombocytopenia in dengue patients from a population in Yogyakarta, Indonesia. Further replication was conducted in the Taiwanese population.

## 2. Materials and Methods

### 2.1. Subjects

This study was a prospective cohort study, enrolling patients who were hospitalized and diagnosed with DF (ICD-10-CM code A90) or DHF (A91) between 2020 and 2022. Participants were recruited from several PKU Muhammadiyah Hospitals in Yogyakarta Province, including the City of Yogyakarta, Sleman Regency, Bantul Regency, and Panembahan Senopati Hospitals (Bantul Regency) in Indonesia. Additionally, participants were recruited from an outbreak diagnosed in Kaohsiung Medical University Hospital, Kaohsiung, Taiwan, in 2015. 

In Indonesia, patients were eligible to participate in this study if they lived in or had traveled to a dengue-endemic area and presented with a fever, a low white cell count, and/or thrombocytopenia. Positive rapid test results (SD Bioline Dengue RDT series) for immunoglobulin M (IgM), IgG, and non-structural protein 1 (NS1) were used to confirm a DENV infection. Patients with positive results for coronavirus disease 2019 (COVID-19) were excluded. Our study protocol was approved by the ethics committee of PKU Muhammadiyah Yogyakarta Hospital (Ref:00101/KT.7.4/III/2021) and the health research ethics committee of the Faculty of Medicine and Health Science, University of Muhammadiyah Yogyakarta (UMY) (Ref: 063/EC-KEPK FKIK UMY/XII/2020). Informed consent was obtained from all study participants before recruitment and sampling.

A clinical diagnosis of dengue (DF, DHF, and DSS) was made by expert physicians following World Health Organization (WHO) 1997 recommendations [20]. Patients with DHF presented with a fever and hemorrhagic manifestations, a low platelet count (<10^5^ cells per mm^3^), and evidence of plasma leakage (such as an increased hematocrit level, pleural effusion, ascites, or hypoproteinemia). Primary dengue infection was defined as the detection of IgM or NS1 results and negative IgG results, while the presence of IgM or NS1 and positive IgG results suggested a secondary dengue infection. Detailed clinical data, including routine laboratory parameters, were collected. The day of illness onset was calculated as the first day of fever.

Study participants from Taiwanese dengue patients were recruited from the Kaohsiung Medical University Chung-Ho Memorial Hospital (KMUH) during the outbreak in 2014–2015. Patients were diagnosed with dengue fever according to the WHO recommendation (1997). Dengue fever was confirmed based on results from RT-PCR and NS1. Clinical data at admission, such as age, gender, and some laboratory values, were extracted from medical records. The study protocol was approved by KMUH Institutional Review Board (Ref: KMUH-IRB-20110451).

### 2.2. DNA Extraction and Genotyping

DNA extractions for Indonesian dengue samples were performed using the QIAamp DNA Blood Mini Kit (Qiagen GmbH, Hilden, Germany) from whole blood of dengue patients according to the manufacturer’s instructions. TaqMan single-nucleotide polymorphism (SNP) genotyping assays were used to determine the genotypes. Furthermore, a polymerase chain reaction (PCR) was performed using Bio-Rad CFX96 (Bio-Rad Laboratories Inc., Hercules, CA, USA). Cycling conditions were as follows: denaturation at 95 °C for 5 min followed by 40 cycles of amplification at 95 °C for 3 s and 60 °C for 40 s. Archived genomic DNA of the Taiwanese cohort from KMUH were genotyped using a TPM 2.0 SNP array. Genotypes of the investigated variants were extracted for analysis.

### 2.3. Statistical Analysis

Statistical analysis was performed using SAS software version 9.4 (SAS, Cary, NC, USA) and R version 4.1.0 (RStudio, 51 Franklin St, Fifth Floor, Boston, MA, USA). The R SNPassoc package was used to analyze associations among dengue severity, clinical phenotypes, and genotypes under genotypic, dominant, recessive, and log-additive models. The significance level was set at *p* < 0.05.

## 3. Results

### 3.1. Demographic and Hematological Values of the Study Population

**Figure 1:**
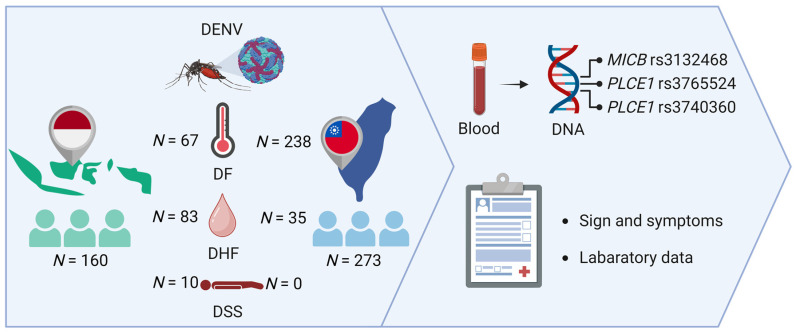
Workflow of the genetic association study (Created with BioRender.com).

In total, 160 dengue patients from the Indonesian population were recruited and genotyped for *MICB* rs3132468 and *PLCE1* rs3740360, rs3765524. Among these patients, 10 (6.25%) were diagnosed with DSS, 83 (51.87%) had DHF, and 67 (41.87%) had DF. In addition, 273 Taiwanese samples were identified as dengue patients, among which 35 (12.82%) were diagnosed with DHF, while none were diagnosed with DSS. A schematic workflow of the study is illustrated in Figure 1, while the demographic characteristics of the study populations are summarized in Table 1.

Based on age, dengue in the Indonesian population is more common in younger individuals, while it predominantly affects adults in the Taiwanese population (*p* < 0.0001). Older age in the Taiwanese population tends to be associated with higher likelihood of developing DHF, and this difference is significant when compared to DF patients (Table S1). In terms of hematological data in the two populations, platelet levels and blood glucose in the Indonesian population had significantly lower mean values than those in the Taiwanese population (platelet level, *p* < 0.0001; blood glucose, *p* = 0.0007). Meanwhile, the mean value of leucocytes in the Indonesian population was higher than in the Taiwanese population (*p* = 0.039).

When comparing baseline characteristics between DF, DHF, and DSS of the Indonesian population, this study found similar characteristics among the groups (Table S2). Importantly, 62.5% of the study population had secondary infections, which were more prevalent in the DSS group (70%) than in the DHF (68.67%) or DF groups (53.73%). A significantly lower platelet count was shown in DSS patients compared to the other two groups (DSS versus DF, *p* = 0.0004; DSS versus DHF, *p* = 0.004). A significant decrease in platelet levels also was also observed in DHF patients in the Taiwanese population compared to DF patients (*p* = 0.0005) (Table S1). Among hematological data, DSS patients presented higher levels of hematocrit and liver enzymes such as aspartate aminotransferase (AST) and alanine aminotransferase (ALT); however, we detected no significant difference in hematocrit or liver enzyme data between DHF and DF patients (Table S3).

### 3.2. Associations of MICB rs3132468, PLCE1 rs3765524, and PLCE1 rs3740360 with Dengue Severity

To gain a deeper understanding of the potential roles of the *MICB* and *PLCE1* genes in the severity of dengue, the numbers of DF, DHF, and DSS patients were compared across *MICB* and *PLCE1* genotypes. Specifically, one SNP on the *MICB* gene (rs3132468), and two SNPs on the *PLCE1* gene (rs3740360 and rs3765524) were selected for genotyping. The gene, variant, position, and allelic frequencies of these three SNPs in various populations are shown in Table 2.

Based on the data in Table 2, the variant of *MICB* rs3132468 was found to have a higher alternative allele frequency in the Taiwanese population than the Indonesian population or other countries. The alternative allele frequency of the variant *PLCE1* rs3765524 was higher in the Indonesian than in the Taiwanese population, while the variant of *PLCE1* rs3740360 in Indonesia showed a higher alternative allele frequency than in other populations. 

Genotype frequencies of *MICB* rs3132468 and *PLCE1* rs3740360 and rs3765524 in the Indonesian population are shown in Table 3 and Table S7, while genotype frequencies of these three variants in the Taiwanese population are presented in Table 4 and Table S8. Results confirmed a significant association of *MICB* rs3132468 with DSS in the Indonesian population compared to DF (log-additive model, *p* = 0.0380). However, there were no significant differences between these three groups in the two variants of *PLCE1* (rs3765524 and rs3740360), suggesting that variants of these genes may not be associated with the severity of dengue in the Indonesian population.

In the replication study, no significant association was found in the *MICB* and *PLCE1* genotypes with DHF in the Taiwanese population (Table 4 and Table S8). 

### 3.3. Associations of MICB rs3132468, PLCE1 rs3765524, and PLCE1 rs3740360 with Platelet Counts

We examined the associations of *MICB* and *PLCE1* genotypes with platelet counts, as an important hematological parameter in dengue. A rapid decrease in platelet count may cause plasma leakage and is suggestive of progression to severe dengue [21]. Among the 160 Indonesian samples, thrombocytopenia (≤10^5^ cells/mm^3^) was observed in 124 patients (77.5%) irrespective of whether they had DF, DHF, or DSS, while only 36 patients had more than 10^5^ cells/mm^3^ platelets during their hospitalization. However, as shown in Table S4, there were no statistically significant differences in platelet counts between the *MICB* and *PLCE1* genotypes.

DHF and DSS are characterized by thrombocytopenia and lower platelet levels. In the Taiwanese dengue cohort, the frequency of thrombocytopenia was lower compared to the Indonesian samples, with only 26.74% (73 patients) experiencing thrombocytopenia during hospitalization. Moreover, there was no significant association between the *MICB* and *PLCE1* genotypes and thrombocytopenia (Table S5). The percentages of thrombocytopenia incidence in the Indonesian and Taiwanese populations were in line with the percentages of patients based on the severity. Furthermore, a higher prevalence of DHF and DSS was observed in the Indonesian population compared to the Taiwanese population, which aligned with the finding that the Indonesian population had lower platelet levels.

### 3.4. Associations of MICB rs3132468, PLCE1 rs3765524, and PLCE1 rs3740360 with Dengue Severity Stratified by Primary or Secondary Dengue

Since the majority of Indonesian patients in this study had a prior infection with another serotype of dengue virus, i.e., secondary dengue (62.5%), we investigated correlations of the *MICB* or *PLCE1* genotypes with the severity of dengue, stratified for primary and secondary infections (Table 5 and Table S6). In this study, we identified a significant association between *PLCE1* rs3740360 and DHF in patients with secondary dengue infection in the dominant model (*p* = 0.0344; OR = 2.5; 95% CI = 1.06–5.89) and the log-additive model (*p* = 0.0250). The results indicated the important role of *PLCE1* rs3740360 in DHF during secondary dengue infection. However, due to unavailable data, no replication analysis was conducted for Taiwanese dengue samples.

## 4. Discussion

In this study, we examined 160 dengue patients from the Indonesian population and 273 patients from the Taiwanese population. According to the demographics, our research indicates a higher prevalence of dengue among younger individuals in the Indonesian population, while it has a more significant impact on adults in the Taiwanese population. Notably, cases of DSS are extremely rare in the Taiwanese population, which may be attributed to the medical support system. Our results further showed the association between *MICB* rs3132468 and DSS, as well as *PLCE1* rs3740360 and DHF in secondary dengue among Indonesian patients. 

This study found that *MICB* rs3132468-C allele is associated with higher risk of DSS, which is consistent with previous research [13]. However, no significant differences were detected in allelic frequencies of *MICB* rs3132468-C and *PLCE1* rs3765524-C between the DF and DHF groups [13]. These findings are consistent with those of the current study in the Indonesian and Taiwanese populations. *MICB* encodes an activating ligand that will be recognized by a NKG2D receptor, a sensor to activate signals for immune cells. The interaction between MICB and NKG2D plays a role in mediating NK cells and eliminating the virus-infected cells [22]. NK cells are known to be robustly activated during the acute phase of DENV infection [15,16]. Previous studies have indicated that the DSS-risk allele (*MICB* rs3132468-C) is linked to reduced mRNA expression of MICB. This connection might influence NK cell response, leading to higher viremia levels and an increased susceptibility to severe dengue [13]. Variants of the *MICB* gene, which is located in a highly polymorphic MHC region, are known to be associated with other infections, such as severe acute respiratory syndrome coronavirus 2 (SARS-CoV-2) and cancer [23,24]. However, it is important to note that this study did not identify any associations between *MICB* and *PLCE1* genotypes when comparing DSS to DHF (as shown in Table S7). DSS, falling within grades III and IV of DHF, shares similar characteristics with DHF, including abnormal hemostasis and plasma leakage. The substantial plasma loss in DHF patients can lead to a critical state of shock, potentially resulting in a poor prognosis and increased mortality [6].

One of the common laboratory findings in dengue is thrombocytopenia. A previous study demonstrated that activated platelets, in response to infectious and cellular interactions with monocytes, contribute to the pathogenesis of dengue [25]. Furthermore, activated platelets were found to be higher in patients with dengue warning signs and severe dengue compared to mild dengue patients [26]. A genetic study reported associations of genetic polymorphisms (the R/R genotype of *FCGR2A* p.R131H and the G/G genotype of *CCL2* c.-2518 A > G) with thrombocytopenia [27]; however, our study did not reveal an association of *MICB* and *PLCE1* variants with thrombocytopenia. Similar findings of a previous study in Vietnam [19] indicated that the functional basis of *MICB* and *PLCE1* variants in thrombocytopenia of dengue patients remains unclear.

Another factor that is known to be related to dengue severity is secondary infection [28,29]. Infection from the first serotype of DENV provides lifelong immunity; however, if a second infection occurs from a heterotypic serotype, it will induce an antibody-dependent enhancement (ADE) phenomenon that can result in severe disease [30]. Polymorphisms in the immune system may have functional significance and may play a role in disease susceptibility or treatment responses [31]. Our study demonstrated that the majority of DHF/DSS patients had previously been infected with a different serotype of DENV. Importantly, a genetic association study showed that a variant of *PLCE1* (rs3740360) is associated with DHF in secondary dengue infection patients. There is no previous study that analyzed the association between *PLCE1* and dengue severity stratified by primary or secondary dengue infections. *PLCE1* is known to regulate small GTPases, such as Ras and RhoA [17]. *PLCE1* is also known to contribute to angiogenesis, which is related to inflammation via the nuclear factor (NF)-κB pathway [32]. In dengue, DENV is known to induce NF-κB activation and present an inflammatory response [33]. Other than that, PLCE1 may contribute to vascular leakage by regulating the Rho family of GTPase [17,18]. Furthermore, RhoA plays a role in the regulation of vascular leakage and endothelial permeability [18]. Plasma leakage is a sign of DHF that can lead to the development of shock (DSS) [6]. Rho GTPase is a subfamily of small GTPases also known to be involved in the pre-entry process of coronavirus infection and endocytosis [34]. In context of the secondary heterologous infection, pre-existing antibodies weakly neutralize the heterologous DENV and increase the viral replication. This ADE phenomenon produces cytokines that induce the vascular leakage [35].

There are several limitations of this study that should be noted. First, our study lacked information related to the level of viremia and the dengue virus serotype that were related to the severity of dengue. Second, the small sample size in both populations might have limited the statistical power. Therefore, further studies with a larger sample size and more clinical information are needed to confirm the associations of *MICB* and *PLCE1* variants with the severity of dengue. This study highlights the value of genetic testing, which may be included in the clinical pathway for specific patients who can be protected from severe dengue.

## 5. Conclusions

In conclusion, we found a higher prevalence of dengue among younger individuals in the Indonesian population, while a more significant impact on adults was identified in the Taiwanese population. The results revealed an association between *MICB* rs3132468 and DSS. *PLCE1* rs3740360 was found to correlate with DHF in secondary dengue patients. However, there was no significant association of *MICB* and *PLCE1* variants in the Taiwanese population.

## Figures and Tables

**Table 1:** Baseline characteristics of the study population.

Characteristic	Indonesian Population (*N* = 160)	Taiwanese Population (*N* = 273)
Age (mean ± SD, years)	17.06 ± 14.51	50.40 ±17.79
Gender		
Male (%)	93 (58.13)	145 (53.11)
Female (%)	67 (41.88)	128 (46.89)
Disease status		
DF (%)	67 (41.87)	238 (87.18)
DHF (%)	83 (51.87)	35 (12.82)
DSS (%)	10 (6.25)	0
Platelets (mean ± SD, 10^3^/mm^3^)	115.50 ± 64.94	139.44 ± 69.27
Hemoglobin (mean ± SD, g/dL)	13.94 ± 2.27	13.87 ± 1.73
Leukocytes (mean ± SD, 10^3^/mm^3^)	4760 ± 4690	4680 ± 2200
Blood glucose (mean ± SD, mg/dL)	118.49 ± 48.80	133.86 ± 46.71

Abbreviations: SD, standard deviation; DF, dengue fever; DHF, dengue hemorrhagic fever; DSS, dengue shock syndrome.

**Table 2:** Basic characteristics of single-nucleotide polymorphisms (SNPs) for the genetic association study.

Gene	Variant	Position ^a^	Ref	Alt	Location	Frequency ^b^					
						African	American	European	Asian	Taiwanese	Indonesian
*MICB*	rs3132468	chr6:31507709	C	T	Intron	0.82	0.81	0.72	0.87	0.92	0.73
*PLCE1*	rs3765524	chr10:94298541	C	T	Missense	0.45	0.21	0.30	0.23	0.19	0.39
*PLCE1*	rs3740360	chr10:94265734	A	C	Intron	0.00	0.06	0.11	0.19	0.17	0.34

^a^ Reference version: GRCh38.p14. ^b^ Frequencies are shown as alternative allelic frequencies.

**Table 3:** Association of *MICB* rs3132468, *PLCE1* rs3765524, *PLCE1* rs3740360, and dengue severity in the Indonesian population.

Genotype	Number of Subjects (*N* = 160)			DF vs. DHF	DF vs. DSS
	DF (%)	DHF (%)	DSS (%)	Log-Additive	Log-Additive
*MICB* rs3132468					
C/C	3 (4.5)	6 (7.2)	2 (20)	0.342	**0.038**
C/T	24 (35.8)	33 (39.8)	5 (50)		
T/T	40 (59.7)	44 (53)	3 (30)		
*PLCE1* rs3765524					
C/C	25 (37.3)	27 (32.5)	5 (50)	0.525	0.506
C/T	33 (49.3)	43 (51.8)	5 (50)		
T/T	9 (13.4)	13 (15.7)	0		
*PLCE1* rs3740360					
A/A	29 (43.3)	30 (36.1)	6 (60)	0.477	0.687
A/C	31 (46.3)	44 (53)	4 (40)		
C/C	7 (10.4)	9 (10.8)	0		

DF: dengue fever; DHF: dengue hemorrhagic fever; DSS: dengue shock syndrome. *p* values < 0.05 are shown in bold

**Table 4:** Association of *MICB* rs3132468, *PLCE1* rs3765524, *PLCE1* rs3740360, and dengue severity in the Taiwanese population.

Genotype	Number of Subjects (*N* = 273)		DF vs. DHF
	DF (%)	DHF (%)	Log-Additive
*MICB* rs3132468			
C/C	1 (0.4)	0	0.824
C/T	36 (15.1)	4 (11.4)	
T/T	201 (84.5)	31 (88.6)	
*PLCE1* rs3765524			
C/C	160 (67.2)	19 (54.3)	0.315
C/T	66 (27.7)	15 (42.9)	
T/T	12 (5.0)	1 (2.9)	
*PLCE1* rs3740360			
A/A	164 (68.9)	21 (60)	0.297
A/C	67 (28.2)	14 (40)	
C/C	7 (2.9)	0	

DF: dengue fever; DHF: dengue hemorrhagic fever.

**Table 5:** Association of *MICB* rs3132468, *PLCE1* rs3765524, *PLCE1* rs3740360, and dengue severity in patients with secondary dengue infection in the Indonesian population.

Genotype	Number of Subjects (*N* = 100)			DF vs. DHF				DF vs. DSS			
	DF (%)	DHF (%)	DSS (%)	Genotypic	Dominant	Recessive	Log- Additive	Genotypic	Dominant	Recessive	Log- Additive
*MICB* rs3132468											
T/T	22 (61.1)	30 (52.6)	2 (28.6)	0.681	0.421	0.559	0.381	0.269	0.111	0.449	0.122
C/T	12 (33.3)	22 (38.6)	4 (57.1)								
C/C	2 (5.6)	5 (8.8)	1 (14.3)								
*PLCE1* rs3765524											
C/C	16 (44.4)	17 (29.8)	4 (57.1)	0.253	0.152	0.204	0.097	1.00	0.538	1.000	1.000
C/T	16 (44.4)	28 (49.1)	3 (42.9)								
T/T	4 (11.1)	12 (21.1)	0								
*PLCE1* rs3740360											
A/A	20 (55.6)	19 (33.3)	5 (71.4)	0.080	**0.034**	0.180	**0.025**	0.780	0.427	1.000	0.780
A/C	14 (38.9)	30 (52.6)	2 (28.6)								
C/C	2 (5.6)	8 (14.0)	0								

DF: dengue fever; DHF: dengue hemorrhagic fever; DSS: dengue shock syndrome. *p* values < 0.05 are shown in bold.

## Data Availability

All data supporting the findings of this study are available within the paper and its Supplementary Information.

## Supplementary Materials

The following supporting information can be downloaded at: https://www.mdpi.com/article/10.3390/diagnostics13213365/s1, Table S1: Baseline characteristics of the Taiwanese population; Table S2: Baseline characteristics of the Indonesian population; Table S3: Hematological values of the Indonesian population; Table S4: Association analysis between *MICB* and *PLCE1* single-nucleotide polymorphisms (SNPs) and platelet counts of the Indonesian population; Table S5: Association analysis between *MICB* and *PLCE1* single-nucleotide polymorphisms (SNPs) and platelet counts of the Taiwanese population; Table S6: Association of *MICB* rs3132468, *PLCE1* rs3765524, *PLCE1* rs3740360, and dengue severity in patients with primary dengue infection in the Indonesian population; Table S7: Association of *MICB* rs3132468, *PLCE1* rs3765524, *PLCE1* rs3740360, and dengue severity in the Indonesian population; Table S8: Association of *MICB* rs3132468, *PLCE1* rs3765524, *PLCE1* rs3740360, and dengue severity in the Taiwanese population.
