# Supplementary material for: Genetic Association Studies of MICB and PLCE1 with Severity of Dengue in Indonesian and Taiwanese Populations
Source: Diagnostics (Basel). 2023 Nov 1;13(21):3365. doi: 10.3390/diagnostics13213365 (PMC10647310; doi:10.3390/diagnostics13213365)
Supplement: Supplementary file 1 [file diagnostics-13-03365-s001.zip › Supplementary_IDN & TW_ Diagnostics template.pdf]

Genetic Association Studies of *MICB* and *PLCE1* with Severity of Dengue in Indonesian and Taiwanese Populations

Imaniar Noor Faridah[1,2], Haafizah Dania[2], Rita Maliza[3], Wan-Hsuan Chou[1], Yen-Hsu Chen*[4], Dyah Aryani Perwitasari[2*], Wei Chiao Chang[1,5,6]*

Table S1. Baseline characteristics of the Taiwanese population

Characteristic	Number of subjects		*p value*
	DF (*N*=238)	DHF (*N*=35)	
Age (mean ± SD, years)	47.97 ± 17.09	66.97 ± 13.09	**<.0001**
Male gender (%)	128 (53.78)	17 (48.57)	0.332
Female gender (%)	110 (46.22)	18 (51.43)	
Platelets (mean ± SD, $10^3/mm^3$)	145.34 ± 66.73	96.97 ± 73.44	**0.0005**

Abbreviations: DF, dengue fever; DHF, dengue hemorrhagic fever; SD, standard deviation

Table S2. Baseline characteristics of the Indonesian population

Characteristic	Number of subjects			*p* value		
	DF (*N*= 67)	DHF (*N*=83)	DSS (*N* =10)	DF vs DHF	DF vs DSS	DHF vs DSS
Age (mean ± SD, years)	19.07 ± 15.63	16.28 ± 14.07	10.05 ± 5.93	0.204	0.078	0.261
Male gender (%)	37 (55.22)	50 (60.24)	6 (60)	0.536	1.000	1.000
Female gender (%)	30 (44.78)	33 (39.76)	4 (40)			
Length of hospitalization (mean ± SD, day)	4.1 ± 1.18	4.39 ± 1.40	4.40 ± 0.97	0.318	0.401	0.828
Secondary infection (%)	36 (53.73)	57 (68.67)	7 (70)	0.060	0.498	1.000
Days of illness (mean ± SD, day)	3.97 ± 1.59	3.93 ± 1.54	3.80 ± 1.23	0.993	0.957	0.954
Platelets (mean ± SD, $10^3/mm^3$)	137.69 ± 78.61	103.79 ± 47.74	64.1 ± 23.62	**0.007**	**0.0004**	**0.004**
Hematocrit (mean ± SD, %)	40.90 ± 5.83	41.40 ± 5.62	43.68 ± 5.62	0.753	0.165	0.297

Abbreviations: DF, dengue fever; DHF, dengue hemorrhagic fever; DSS, dengue shock syndrome; SD, standard deviation

Table S3. Hematological values of Indonesian population

Variables (units)	N	Mean of variables ± SD				*p* value		
		All dengue	DF	DHF	DSS	DF vs DHF	DF vs DSS	DHF vs DSS
Leucocytes (/mm^3)	140	4760 ± 4690	4560 ± 2830	4780 ± 5900	5710 ± 3000	0.533	0.173	0.072
Hemoglobin (g/dL)	142	13.94 ± 2.27	13.97 ± 2.18	13.82 ± 2.38	14.67 ± 1.92	0.553	0.307	0.214
ALT (U/L)	32	102.68 ± 120.35	83.26 ± 68.41	106.16 ± 131.76	279.50 ± 365.57	0.982	0.954	0.914
AST (U/L)	32	165.24 ± 218.70	143.81 ± 187.92	162.88 ± 230.77	391.40 ± 470.08	0.613	0.775	0.591
Blood glucose (mg/dL)	55	118.49 ± 48.80	125.82 ± 64.15	108.22 ± 22.47	126.20 ± 39.65	0.704	0.659	0.352

SD: standard deviation; DF: dengue fever; DHF: dengue hemorrhagic fever; ALT: alanine aminotransferase; AST: aspartate aminotransferase.

Table S4. Association analysis between *MICB* and *PLCE1* single nucleotide polymorphisms (SNP)s and platelet count of Indonesian population (n=160)

Gene / Variant	Genotype	Platelet count (10^3/mm^3)		Genotypic	Dominant	Recessive	Log-Additive
		≤ 100 (%)	> 100 (%)	*p* value	*p* value	*p* value	*p* value
MICB rs3132468	CC	9 (7.3)	2 (5.6)	0.649	0.355	0.716	0.371
	CT	50 (40.3)	12 (33.3)				
	TT	65 (52.4)	22 (61.1)				
PLCE1 rs3765524	CC	46 (37.1)	11 (30.6)	0.753	0.467	0.978	0.596
	CT	61 (49.2)	20 (55.6)				
	TT	17 (13.7)	5 (13.9)				
PLCE1 rs3740360	AA	54 (43.5)	11 (30.6)	0.364	0.157	0.803	0.238
	AC	58 (46.8)	21 (58.3)				
	CC	12 (9.7)	4 (11.1)				

Table S5. Association analysis between *MICB* and *PLCE1* single nucleotide polymorphisms (SNP)s and platelet count of Taiwanese Population (n=271)

Gene / Variant	Genotype	Platelet count (10^3/mm^3)		Genotypic	Dominant	Recessive	Log-Additive
		≤ 100 (%)	> 100 (%)	*p* value	*p* value	*p* value	*p* value
MICB rs3132468	CC	1 (1.4)	0	0.359	0.986	0.269	0.358
	CT	10 (13.7)	30 (15.2)				
	TT	62 (84.9)	168 (84.8)				
PLCE1 rs3765524	CC	52 (71.2)	127 (64.1)	0.543	0.269	0.744	0.302
	CT	18 (24.7)	61 (30.8)				
	TT	3 (4.1)	10 (5.1)				
PLCE1 rs3740360	AA	53 (72.6)	132 (66.7)	0.532	0.347	0.415	0.285
	AC	19 (26.0)	60 (30.3)				
	CC	1 (1.4)	6 (3.0)				

Table S6. Association of *MICB* rs3132468, *PLCE1* rs3765524, *PLCE1* rs3740360 and dengue severity in patients with primary dengue infection in Indonesian population

Genotype	Number of subjects (*N*=60)			DF vs DHF				DF vs DSS				DHF vs DSS			
	DF (%)	DHF (%)	DSS (%)	Genotypic	Dominant	Recessive	Log-Additive	Genotypic	Dominant	Recessive	Log-Additive	Genotypic	Dominant	Recessive	Log-Additive
MICB rs3132468															
T/T	18 (58.1)	14 (53.8)	1 (33.3)												
C/T	12 (38.7)	11 (42.3)	1 (33.3)	0.948	0.749	0.899	0.747	0.267	0.410	0.109	0.156	0.318	0.497	0.132	0.203
C/C	1 (3.2)	1 (3.8)	1 (33.3)												
PLCE1 rs3765524															
C/C	9 (29.0)	10 (38.5)	1 (33.3)												
C/T	17 (54.8)	15 (57.7)	2 (66.7)	0.265	0.452	0.114	0.186	1.000	0.877	1.000	1.000	1.000	0.861	1.000	1.000
T/T	5 (16.1)	1 (3.8)	0												
PLCE1 rs3740360															
A/A	9 (29.0)	11 (42.3)	1 (33.3)												
A/C	17 (54.8)	14 (53.8)	2 (66.7)	0.227	0.295	0.114	0.123	1.000	0.877	1.000	1.000	1.000	0.762	1.000	1.000
C/C	5 (16.1)	1 (3.8)	0												

DF: Dengue Fever; DHF: Dengue Hemorrhagic Fever; DSS : Dengue Shock Syndrome

Table S7. Association of *MICB* rs3132468, *PLCE1* rs3765524, *PLCE1* rs3740360 and dengue severity in Indonesian population

Genotype	Number of subjects (*N*=160)			DF vs DHF			DF vs DSS			DHF vs DSS			
	DF (%)	DHF (%)	DSS (%)	Genotypic	Dominant	Recessive	Genotypic	Dominant	Recessive	Genotypic	Dominant	Recessive	Log-additive
MICB rs3132468													
T/T	40 (59.7)	44 (53)	3 (30)										
C/T	24 (35.8)	33 (39.8)	5 (50)	0.629	0.411	0.475	0.115	0.076	0.113	0.275	0.164	0.228	0.108
C/C	3 (4.5)	6 (7.2)	2 (20)										
PLCE1 rs3765524													
C/C	25 (37.3)	27 (32.5)	5 (50)										
C/T	33 (49.3)	43 (51.8)	5 (50)	0.811	0.540	0.700	0.506	0.447	0.595	0.373	0.282	0.347	0.373
T/T	9 (13.4)	13 (15.7)	0										
PLCE1 rs3740360													
A/A	29 (43.3)	30 (36.1)	6 (60)										
A/C	31 (46.3)	44 (53)	4 (40)	0.662	0.373	0.937	0.687	0.322	0.585	0.316	0.149	0.589	0.316
C/C	7 (10.4)	9 (10.8)	0										

DF: dengue fever; DHF: dengue hemorrhagic fever; DSS: dengue shock syndrome

Table S8. Association of *MICB* rs3132468, *PLCE1* rs3765524, *PLCE1* rs3740360 and dengue severity in Taiwanese population

Genotype	Number of subjects (*N*=273)		DF vs DHF		
	DF (%)	DHF (%)	Genotypic	Dominant	Recessive
MICB rs3132468					
T/T	201 (84.5)	31 (88.6)			
C/T	36 (15.1)	4 (11.4)	0.824	0.511	1.000
C/C	1 (0.4)	0			
PLCE1 rs3765524					
C/C	160 (67.2)	19 (54.3)			
C/T	66 (27.7)	15 (42.9)	0.194	0.139	0.545
T/T	12 (5)	1 (2.9)			
PLCE1 rs3740360					
A/A	164 (68.9)	21 (60)			
A/C	67 (28.2)	14 (40)	0.297	0.299	0.600
C/C	7 (2.9)	0			

DF: dengue fever; DHF: dengue hemorrhagic fever; DSS: dengue shock syndrome
